# Fabrication of Silver Nanoparticles Dispersed in Palm Oil Using Laser Ablation

**DOI:** 10.3390/ijms11114764

**Published:** 2010-11-22

**Authors:** Reza Zamiri, Azmi Zakaria, Hossein Abbastabar Ahangar, Amir Reza Sadrolhosseini, Mohd Adzir Mahdi

**Affiliations:** 1 Department of Physics, Faculty of Science, Universiti Putra Malaysia, 43400 UPM Serdang, Selangor, Malaysia; E-Mails: zamiri.r@gmail.com (R.Z.); amir1348@gmail.com (A.R.S.); 2 Advanced Materials and Nanotechnology Laboratory, Institute of Advanced Technology, Universiti Putra Malaysia, 43400 UPM Serdang, Selangor, Malaysia; 3 Department of Chemistry, Faculty of Science, Universiti Putra Malaysia, 43400 UPM Serdang, Selangor Darul Ehsan, Malaysia; E-Mail: abbastabar@gmail.com; 4 Wireless and Photonics Networks Research Center, Faculty of Engineering, Universiti Putra Malaysia, 43400 UPM Serdang, Selangor, Malaysia; E-Mail: mdadzir@eng.upm.edu.my

**Keywords:** silver nanoparticles, laser ablation, palm oil

## Abstract

In this study we used a laser ablation technique for preparation of silver nanoparticles. The fabrication process was carried out by ablation of a silver plate immersed in palm oil. A pulsed Nd:YAG laser at a wavelength of 1064 nm was used for ablation of the plate at different times. The palm coconut oil allowed formation of nanoparticles with very small and uniform particle size, which are dispersed very homogeneously within the solution. The obtained particle sizes for 15 and 30 minute ablation times were 2.5 and 2 nm, respectively. Stability study shows that all of the samples remained stable for a reasonable period of time.

## 1. Introduction

The unique properties of noble metals’ nanoparticles (NPs) that differ from bulk materials have strongly excited researchers’ attention over recent years. Actually it was found that the properties of material with the dimension of nano-meter depend on the shape, size and surrounding medium of NPs [1,2]. As a result of this feature, NPs have many applications in many research areas such as life environments, optics and catalysis [3,4]. Among the many applications attributed to silver nanoparticles (Ag-NPs), nanocomposite fabrications and antibacterial applications are the most important [5–7].

**Figure 1.**
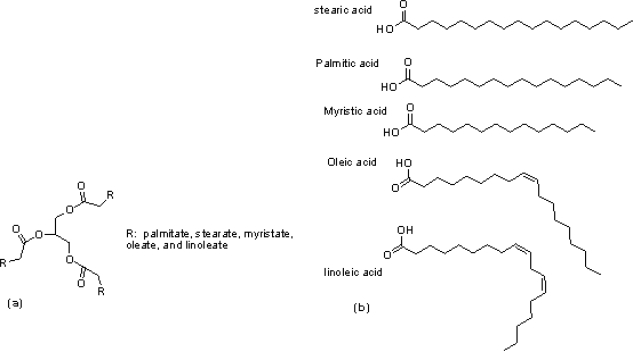
(**a**) The general chemical structure of palm oil; (**b**) the chemical structure of fatty acids in palm oil.

A big challenge in the synthesis of NPs is agglomeration and collapsing their colloidal via precipitation or flocculation due to thermodynamic principals. This tendency can be inhibited by stabilization of NPs with chemical species. This provides obstacles for NPs agglomeration through charge stabilization and steric stabilization. Therefore, much effort has been undertaken to develop different ligands as colloidal stabilizers [8,9]. Recently, synthesis of NPs using fatty acids (oleic and lauric acid) as stabilizers has been reported [10,11]. These organic compounds are amphiphilic molecules with polar carboxylic group, which is able to coordinate to NPs and non polar long carbon chains that prevent NPs agglomeration through steric repulsion. As an example of this kind of preparation, recently, researchers reported vegetable oils as stabilizing agents for preparation of NPs [12]. One of the four commercial vegetable oils traded on the world market is palm oil which is cheaper than canola, soybean, and rapeseed oil. It is very stable towards oxidation due to a maximum proportion of saturates compared to all vegetable oils [13]. Palm oil contains palmitic 44.3%, stearic 4.6%, myristic 1%, oleic 38.7% and linoleic 10.5% (Figure 1a). The presence of long hydrocarbon chains and polar ester bond make it a good choice for stabilizing NPs (Figure 1b).

In this work, we prepared Ag-NPs in palm oil to test the ability of this vegetable oil as a stabilizing and/or dispersing agent. To the best of our knowledge, so far no researchers use palm oil for the preparation of NPs using this technique.

## 2. Experimental

### 2.1. Materials

Palm oil and Ag plate were obtained from Fluka and Sigma Alderic, respectively. Ag plate was purified for 30 min using a sonicated bath. The pulsed Q-Switched Nd:YAG laser (Brilliant), with pulse duration of 5 ns and 10 Hz repetition rate at its original wavelength (1064 nm), was used in this experiment.

### 2.2. Synthesis

**Figure 2.**
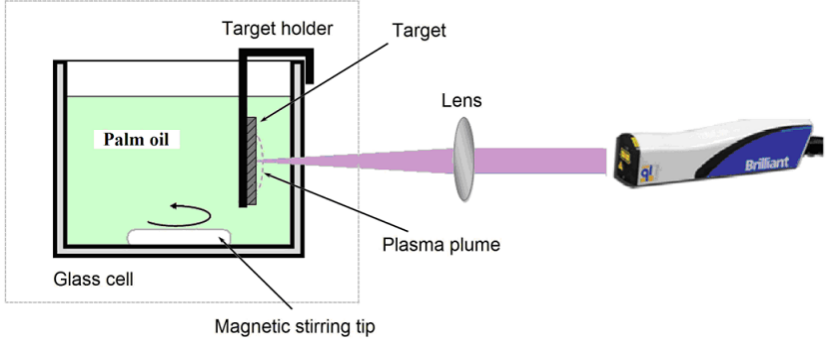
The laser ablation experimental set up.

For the preparation of Ag-NPs, we irradiated the metal silver plate in palm oil by laser under different irradiation times (5, 10, 15, and 30 min). As shown in Figure 2, the metal plate (>99.99%) was placed in a glass cell which was filled with a 10 mL of palm oil. A laser beam was focused on the silver target using a lens with focal length of 250 mm. During the ablation process the solution was stirred magnetically to disperse the produced NPs. The laser output power was 360 mJ/pulse.

### 2.3. Characterization

The prepared Ag-NPs were characterized using an UV-Visible double beam photospectrometer (Shimadzu) and Transmission Electron Microscopy (TEM, Hitachi H-7100). We also calculated the volume fraction of NPs inside the solution using the following equation:

(1) $$V=\frac{V_{S}}{V_{S}+V_{L}}$$

where *V_L_* is the oil volume and *V_S_* = *m*/*ρ* is the volume of the particles where *ρ* is the mass density of the silver and *m* is the particles mass dispersed in the oil. An atomic absorption spectrometer (S Series) has been used to measure *m*. The obtained volume fractions of samples are 0.007 × 10^−6^, 0.010 × 10^−6^, 0.017 × 10^−6^ and 0.028 × 10^−6^ respectively after 5, 10, 15 and 30 min ablation times.

## 3. Results and Discussion

**Figure 3.**
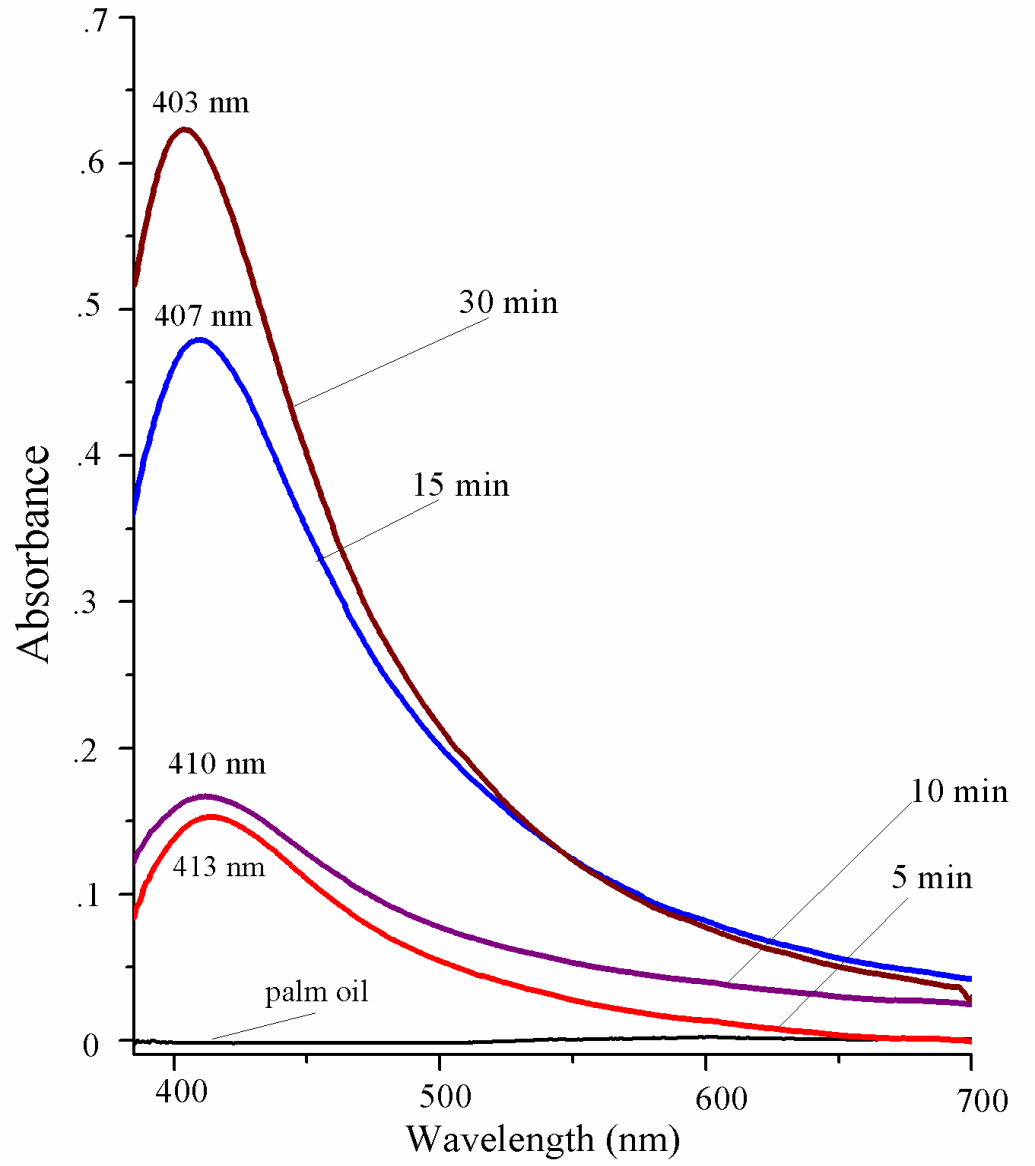
UV-Visible absorption spectra of samples containing Ag-NPs prepared for different ablation times in palm oil.

Figure 3 shows UV-Visible absorption spectrum of samples precisely after the laser ablation process. The peak at 400 nm is a specific peak of Ag-NPs, and can therefore confirm the formation of NPs in the solution. It can also be seen from the figure that by increasing the ablation time from 5 to 30 minutes, the formation efficiency and absorbance peaks are increased and shift to higher energies. According to Mie theory the blue shift in spectra corresponds to the decrement in particle size.

We attribute this decrement of particle size for longer ablation times to be due to interaction between generated particles and laser light. Many metal particles were generated on the path of the incidental laser light. As a result of the interaction between the laser light and particles, the large particles fragment and become smaller. The efficiency of fragmentation increases with increasing ablation time; therefore the obtained particles at longer ablation times are smaller [14].

**Figure 4.**
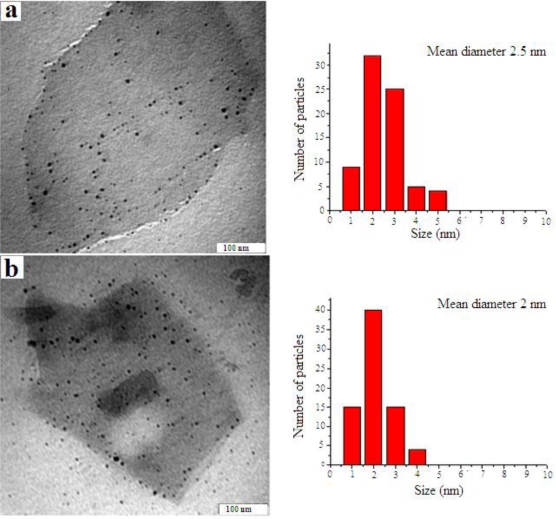
TEM images and typical of statistical graphs for Ag-NPs in palm oil under (**a**) 15 and (**b**) 30 min ablation times.

Figure 4 shows electron micrographs and corresponding size distributions of the samples that were prepared by laser ablation under 15 and 30 min irradiation times; obtaining an average particle size of 2.5 and 2 nm, respectively. The TEM pictures also show well dispersed, as well as non-agglomerated, Ag-NPs with spherical morphology.

The other interesting feature that we observed for all samples was their stability over time. The measured UV-Visible spectrum for sample prepared under 30 min ablation times did not show any specific reduction in peak intensity and also change in full width at half maximum (FWHM) after around two months. This can confirm the stability of the samples. Therefore the obtained results show the ability of palm oil as a stabilizer and size controller.

These abilities can be explained by the competition between the rapid formation of initial silver particle and a consecutive growth of the particle, with termination of this growth due to palm oil molecules capping the particle. If we consider the chronological order of particle formation, instantly after the ablation of the metal plate, a dense cloud of silver atoms is formed over the ablation spot. The interaction between these silver atoms is much stronger than the interaction between silver atoms and solvent molecules, in this case palm oil molecules. Therefore the silver atoms rapidly agglomerate. This process continues until all silver atoms in the close vicinity are consumed. The formation of embryonic silver particles in a region that is already empty of silver atoms results. Even after rapid growth ceases, provision of silver atoms from outside of the region through diffusion, causes the particles to grow slowly. On the other hand, palm oil molecules can, however, adsorb the particles and terminate this growth by capping the particles surface [15].

**Figure 5.**
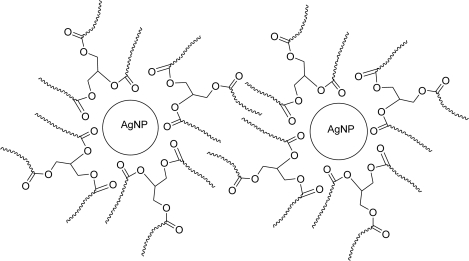
Schematic representation of steric stabilization of Ag-NPs.

The mechanism of adsorption and capping of Ag-NPs by palm oil can be explained through coordination of carbonyl bond, *i.e*., electron transfer from C=O to Ag-NP [16]. On the other hand, the long chain hydrocarbons prevent NPs agglomeration by steric repulsions (Figure 5). The motion of adsorbed molecules is restricted in the interparticle space, which leads to a decrease in entropy [17].

## 4. Conclusions

We have successfully fabricated Ag-NPs in palm oil using a laser ablation technique. The preparation has been carried out for different ablation durations. The obtained particle sizes were 2.5 and 2 nm for 15 and 30 min ablation times, respectively. The results showed that the NPs in the palm oil are well dispersed and non-agglomerated and show stability over a long period of time. All of these observations were attributable to the specific features of palm oil molecules which enable adsorption and capping of NPs.
